# Subcellular redistribution and sequential recruitment of macromolecular components during SGIV assembly

**DOI:** 10.1007/s13238-016-0292-3

**Published:** 2016-07-18

**Authors:** Yongming Yuan, Yunhan Hong

**Affiliations:** Department of Biological Sciences, National University of Singapore, Science Drive 4, Singapore, 117543 Singapore

**Keywords:** ES cell, medaka, *orf088*, SGIV, viral assembly

## Abstract

Virus infection consists of entry, synthesis of macromolecular components, virus assembly and release. Understanding of the mechanisms underlying each event is necessary for the intervention of virus infection in human healthcare and agriculture. Here we report the visualization of Singapore grouper iridovirus (SGIV) assembly in the medaka haploid embryonic stem (ES) cell line HX1. SGIV is a highly infectious DNA virus that causes a massive loss in marine aquaculture. Ectopic expression of VP88GFP, a fusion between green fluorescent protein and the envelope protein VP088, did not compromise the ES cell properties and susceptibility to SGIV infection. Although VP88GFP disperses evenly in the cytoplasm of non-infected cells, it undergoes aggregation and redistribution in SGIV-infected cells. Real-time visualization revealed multiple key stages of VP88GFP redistribution and the dynamics of viral assembly site (VAS). Specifically, VP88GFP entry into and condensation in the VAS occurred within a 6-h duration, a similar duration was observed also for the release of VP88GFP-containing SGIV out of the cell. Taken together, VP088 is an excellent marker for visualizing the SGIV infection process. Our results provide new insight into macromolecular component recruitment and SGIV assembly.

**Electronic supplementary material:**

The online version of this article (doi:10.1007/s13238-016-0292-3) contains supplementary material, which is available to authorized users.

## INTRODUCTION

In human, viral infection causes severe infectious diseases and death such as SARS and H5N1 (Li et al., 2004; Ge et al., 2013). In animal husbandry, viral infection perhaps represents the greatest challenge that results in a massive or complete loss in fish and shellfish aquaculture (Walker and Winton, 2010). Viral infection involves several major steps of viral entry, replication, assembly and release (Dimmock et al., 2007). Understanding of these processes and underlying molecular mechanisms is necessary to develop antiviral drugs and approaches in human healthcare and animal production.

More than 25 virus species have been described in diverse fish species of aquaculture importance, 9 of which are listed by the Office of International Epizootic as highly infectious and notifiable viruses (http://www.oie.int). The highly infectious Singapore grouper iridovirus (SGIV) was first isolated in Singapore from the diseased brown spotted grouper (*Epinephelus tauvina*) as a novel member of genus *Ranavirus* in the family *Iridoviridae* (Qin et al., 2001). In natural and farmed habitats, SGIV infection causes serious systemic diseases and massive death in wild and farmed groupers as well as many other marine teleosts (Qin et al., 2003). In cell cultures, SGIV infection induces paraptosis in its natural host species but apoptosis in non-natural hosts (Huang et al., 2011; Yuan et al., 2013). The SGIV genome is a circular double-stranded DNA of 140,131 bp and predicts 162 protein-encoding genes or open reading frames (ORFs) (Song et al., 2004). According to the timing of expression after entry into the host cells, SGIV genes fall into three major groups, which are immediate-early (IE), delay early (DE) and late groups (Williams et al., 2005; Teng et al., 2008). Generally speaking, IE and DE genes are thought to encode regulatory proteins and key catalytic enzymes involved in cellular immune response, cell-cycle control and apoptosis, whereas late genes often code for viral structural proteins that participate in virion formation in a particular cellular compartment called the viral assembly site (VAS) (Chen et al., 2006; Xia et al., 2009). In order to study the viral protein subcellular distribution, the SGIV IE genes of *orf086* and *orf162* (Xia et al., 2009; Xia et al., 2010), DE gene *orf136* (Huang et al., 2008) and late gene *orf019* (Huang et al., 2013) were overexpressed in host cells with fused EGFP tag respectively. It revealed that the forced expressed protein encoded by these IE and DE genes was distributed in the cytoplasm, but the protein encoded by the late gene condensed in the VAS at the late stage of SGIV infection. These distribution patterns were further verified with immunofluorescent staining, which demonstrated the reliability of using the ectopically expressed fusion protein to analyze the subcellular location of the viral protein.

We make use of medaka (*Oryzias latipes*) as a model to study virus-host interactions in fish. Medaka is a laboratory fish and holds many genetic resources and toolboxes to study their functions in cellular processes and viral infection. This fish has many stem cell lines (Hong et al., 1996, 1998; Hong et al., 2004b; Yi et al., 2009), which are susceptible to infection by aquaculture-important viruses such as the spring viremia of carp virus (SVCV), viral nervous necrosis virus (VNNV) and SGIV (Yuan et al., 2013). More specifically, medaka has given rise to haploid embryonic stem (ES) cell lines that are capable of whole animal production by semicloning (Yi et al., 2009) and susceptible to SVCV, VNNV and SGIV (Yuan et al., 2013). Therefore, medaka represents a unique organism for haploid genetic screening for host factors that viruses use for infection in fish, as has recently been demonstrated for the identification of human genes essential for influenza A infection in a near-haploid cell line (Carette et al., 2009).

SGIV assembly in VAS is a key step in the viral infection cycle. The assembled viruses are subsequently matured and released as infectious pathogens. This study was aimed to identify a molecule that is suitable for visualizing dynamic processes of SGIV assembly and release in a living cell. The SGIV late gene encoded protein VP088 was identified as a putative myristylated envelope protein (Zhou et al., 2011). We overexpressed this protein in host cells and show that the transgene expression of VP88GFP, a fusion between VP088 and green fluorescent protein (GFP), does not compromise the ES cell properties and susceptibility to SGIV. More importantly, VP88GFP shows the dynamic distribution in subcellular compartments. Specifically, the fusion protein disperses evenly in the cytoplasm and undergoes aggregation and redistribution after SGIV infection, which allows for real-time visualization of VAS dynamics in living cells. These results suggest that VP088 plays an important role in SGIV assembly and represents a suitable fusion partner for the production of GFP-tagged recombinant SGIV towards screening for drugs and host factors that control SGIV infection in medaka haploid ES cells.

## RESULTS

### Production of transgenic HX1 cells

**Figure 1:**
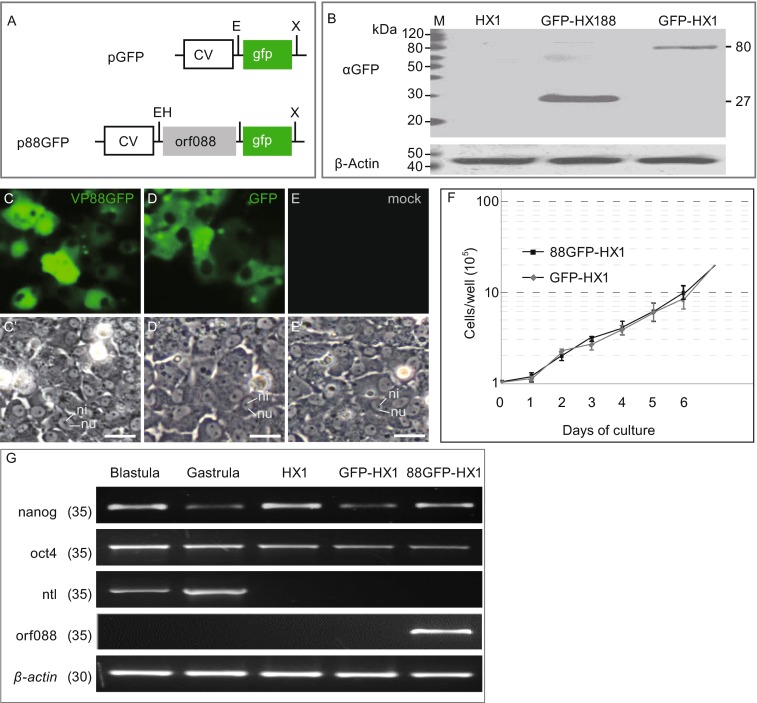
**VP88GFP expression retains the ES cell properties**. HX1 cells at 48 h post mock, pGFP and p88GFP transfection were analyzed by microscopy. (A) Schematic structures of pGFP and p88GFP, which express GFP alone and the fusion protein VP88GFP between VP088 and GFP. CV, the human cytomegalovirus early promoter; E, H and X, sites for *Eco*RI, *Hin*dIII and *Xho*I. (B) Western blot analysis. Cell lysate from HX1, GFP-HX1 and 88GFP-HX1 were analyzed by using αGFP. GFP is seen as a 27-kDa band, and VP88GFP as an 80-kDa band. β-Actin was detected by using as a loading control (*β-actin*). (C–E’) Fluorescent and phase-contrast micrographs. ni, nucleolus; nu, nucleus. Scale bars, 10 µm. (F) The growth of GFP-HX1 and 88GFP-HX1 cells. Data are means ± S.D. (error bars) from two independent experiments in triplicates. (G) RT-PCR analysis of gene expression. Blastula and gastrula embryos were used as the positive control. Numbers of PCR cycles are given in parenthesis

The SGIV VP088 encoded by *orf088* was chosen as a marker to visualize viral infection in cell culture. This protein is expressed late during SGIV infection in grouper cells and represents a putative SGIV envelope protein (Zhou et al., 2011), which is conserved in several iridoviruses (Fig. S1). The gene of *orf088* was inserted in frame ahead of green fluorescent protein (GFP) in pGFP, resulting in p88GFP (Fig. 1A), which expresses VP88GFP, a fusion protein of 746 amino acid residues between VP088 and GFP (Fig. S2).

The medaka haploid ES cell line HX1 was chosen as a cellular model to study the SGIV infection process, because it offers a unique opportunity for genetic screening for molecular virus-host interactions and readily detectable cellular properties such as the ES cell phenotype, pluripotency expression and stable growth (Yi et al., 2009, 2010). The vectors of pGFP and p88GFP were separately transfected into HX1, producing stable transgenic clones of 88GFP-HX1 and GFP-HX1. On a Western blot analysis, GFP was detected as a band of about 27-kDa, and VP88GFP as a band of about 80-kDa (Fig. 1B).

### VP088 expression retains cellular properties

Upon transgenic expression in HX1 cells, VP88GFP was evenly distributed in the cytoplasm (Fig. 1C), which is not different from GFP (Fig. 1D). Expression of either VP88GFP or GFP did not alter the ES cell phenotype, since the transgenic cells displayed a round shape, little cytoplasm and prominent nuclei with large nucleoli (Fig. 1C’–E’), as has been reported for medaka diploid ES cell lines (Hong et al., 1996) and haploid ES cell lines including HX1 (Yi et al., 2009). Furthermore, stable growth of VP88GFP-expressing cells 88GFP-HX1 was similar to that of GFP-expressing cells GFP-HX1 (Fig. 1F). Moreover, VP88GFP expression did not change pluripotency expression in HX1 cells, as the parental HX1 cells and their derivatives transgenic for VP88GFP and GFP all expressed pluripotency genes *nanog* and *oct4*, but not *ntl*, a differentiation marker (Fig. 1G).

### Dynamics of subcellular distribution of VP88GFP

**Figure 2:**
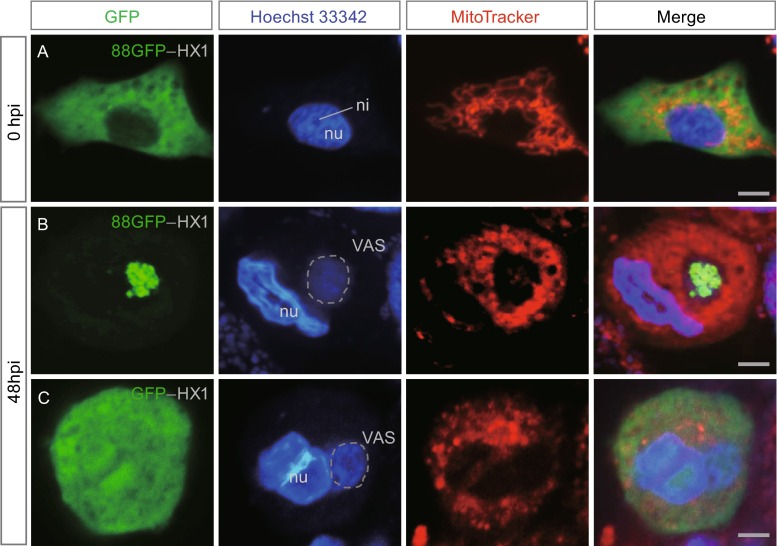
**Subcellular distribution of VP88GFP**. HX1 cells were stained with Hoechst 33342 (blue) plus MitoTracker (red) and analyzed by fluorescent microscopy at indicated hpi with SGIV. (A and B) 88GFP-HX1 cells before (A) and 48 h post SGIV infection (B) showing prominent nucleus (nu), well organized mitochondria and wide distribution of VP88GFP (green) in cytoplasm in the absence of SGIV infection (A) and VP88GFP localization in the virus assembly site (VAS; circle), in which most of the mitochondria are excluded. (C) GFP-HX1 cells at 48 hpi with SGIV, showing the lack of GFP localization in VAS. nu, nucleus; ni, nucleolus. Scale bars, 5 µm

As mentioned above, VP88GFP exhibited an even distribution in the cytoplasm, which became more evident at large magnification, where VP88GFP was found to be distributed almost evenly in the cytoplasm (Fig. 2A), and the virus inoculation procedure (at 0 hpi) did not alter the even distribution. After SGIV infection, VP88GFP altered subcellular distribution depending on intervals post infection. At 48 hpi, VP88GFP was highly condensed in the VAS, which resided close to the nucleus and was intensely stained by Hoechst 33342 due to the viral DNA (Fig. 2B and 2C). For comparison, GFP and mitochondria did not exhibit redistribution and localization (Fig. 2A–C). Taken together, VP88GFP shows uniform cytoplasmic distribution on its own and undergoes redistribution into the VAS in SGIV-infected HX1 cells.

**Figure 3:**
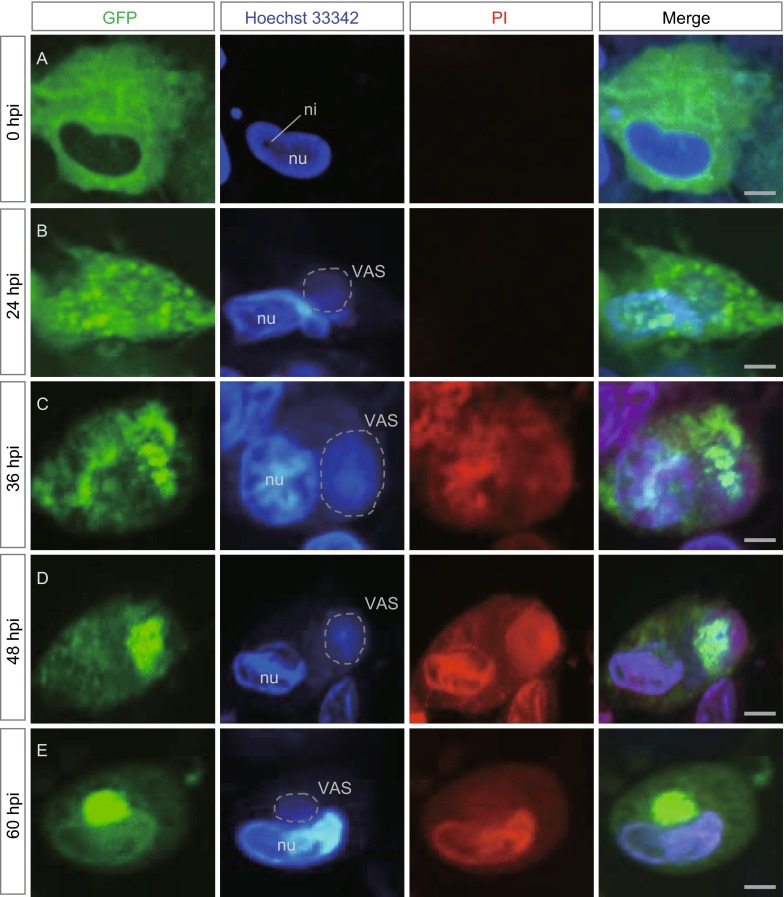
**Dynamics of VP88GFP distribution and cell death**. 88GFP-HX1 cells were infected with SGIV, stained for nuclei with Hoechst 33342 (blue for live cells) plus propidium iodide (PI; red for dead/dying cells) and analyzed by fluorescent microscopy. (A) 88GFP-HX1 cells, showing normal nuclei and even distribution of VP88GFP. (B) 88GFP-HX1 cells at 24 hpi with SGIV, showing viral DNA concentration within the VAS (blue; circle) near the nucleus and VP88GFP aggregation. (C) 88GFP-HX1 at 36 hpi with SGIV, showing VP88GFP distribution into the VAS and cellular death with PI-stainable nuclei (red). (D and E) 88GFP-HX1 at 48 and 60 hpi with SGIV, showing VP88GFP concentration (D) and condensation (E) in the VAS. nu, nucleus; ni, nucleolus. Scale bars, 3 µm

**Figure 4:**
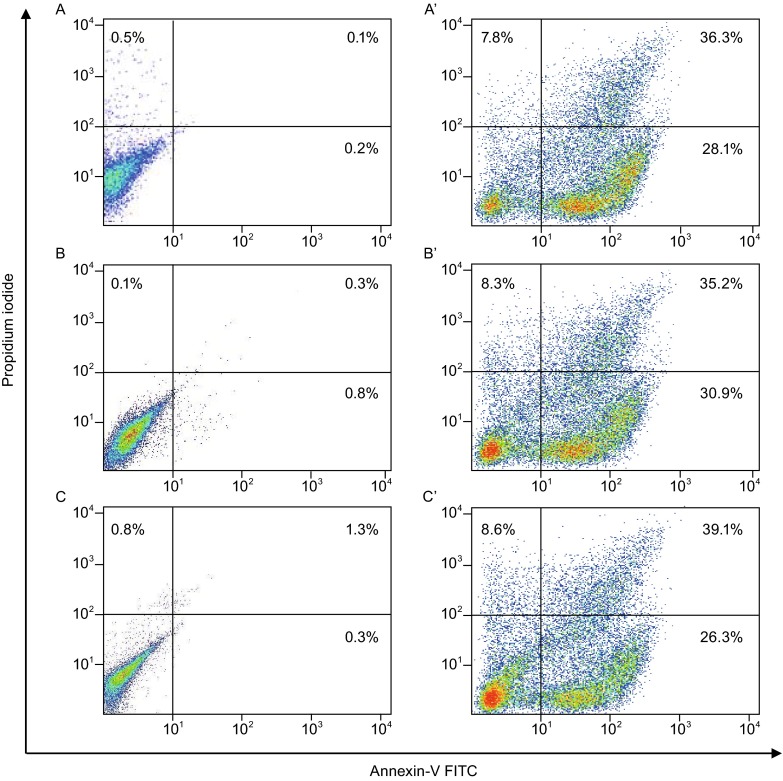
**SGIV causes medaka cell apoptosis**. HX1, GFP-HX1and 88GFP-HX1cells at 48 hpi with SGIV were stained with Annexin-V pacific blue and PI for flow cytometric analysis. Double negative indicates viable cell population, Annexin positive indicates the apoptotic population, PI positive or PI-Annexin double positive indicates the necrotic population. (A–C) HX1, GFP-HX1and 88GFP-HX1cells without SGIV infection. (A’–C’) HX1, GFP-HX1and 88GFP-HX1cells infected with SGIV at 48 hpi, showing increased percentage of apoptotic and necrotic cells. Early apoptotic cells exhibit Annexin (+)/PI (−); late apoptotic cells exhibit Annexin (+)/PI (+); necrotic cells are Annexin (−)/PI (+)

SGIV infection elicits cell death (Huang et al., 2011; Yuan et al., 2013). It has remained unknown when cell death occurs during SGIV infection. We examined this issue by using fluorescent nuclear dyes Hoechst 33342 and PI. Hoechst 33342 stains both live and dying/dead cells, while PI stains dying/dead cells only. HX1 cells shortly after SGIV infection were positive for Hoechst 33342 but negative for PI (Fig. 3A), suggesting they were living cells as expected. A similar staining was seen also in cells until 24 hpi with SGIV, when VP88GFP formed aggregates and the VAS became visible as a Hoechst 33342-stainable DNA-rich area near the nucleus (Fig. 3B), implying cellular viability at this stage. Interestingly, VP088 remained predominantly in the cytoplasm when VAS was already visible, which demonstrates that VAS formation is independent on VP088. Apparently, the cells became positive for both Hoechst 33342 and PI at 36 hpi, when some VP88GFP aggregates were seen in the VAS (Fig. 3C), indicating the onset of cell death detectable by this staining procedure. Staining with Hoechst 33342 and PI became more confined to the nucleus at 48 (Fig. 3D) and 60 hpi (Fig. 3E). By flow cytometry analyses, SGIV infection caused massive cell death in HX1 cells, with a minority of dead cells being necrotic (7.8%) and a majority being apoptotic (64.4%). Similar values were obtained in GFP-HX1 (8.3% and 66.1%) and 88GFP-HX1 cells (8.6% and 65.4%) (Fig. 4), which indicates that the VP88GFP expression does not alter the cell death profile and pathways. Taken together, VP88GFP localizes into the VAS of SGIV-infected cells and the overexpressed VP88GFP does not change the cell death type of host.

### Visualization of viral assembly

**Figure 5:**
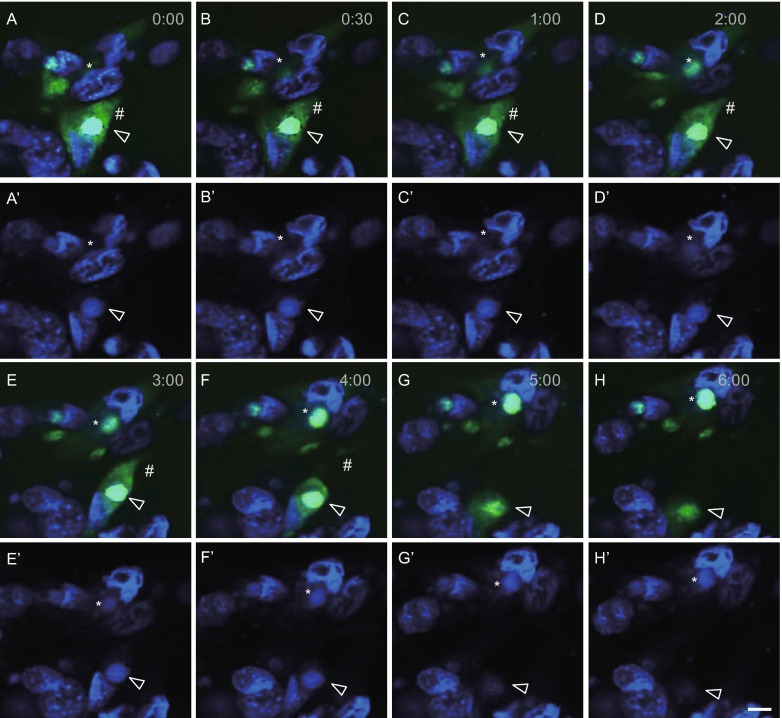
**Visualization of viral assembly site formation and disassembly**. 88GFP-HX1 cells were infected with SGIV, stained for nuclei and VAS with Hoechst 33342 (blue). Time-lapse images represented stages of VP88GFP redistribution at intervals from 48 hpi onwards (For more details, see Movie S1 in the supplemental material). Upper panel, merged signals of VP88GFP and Hoechst 33342, showing colocalization of VP88GFP and VAS. Lower panel, signal of Hoechst 33342 showing the nuclei and VAS. Asterisk, the ongoing formation of VAS; triangle, earlier formed VAS containing VP88GFP; hash, release of cellular components containing VP88GFP. Scale bar, 5 µm

**Figure 6:**
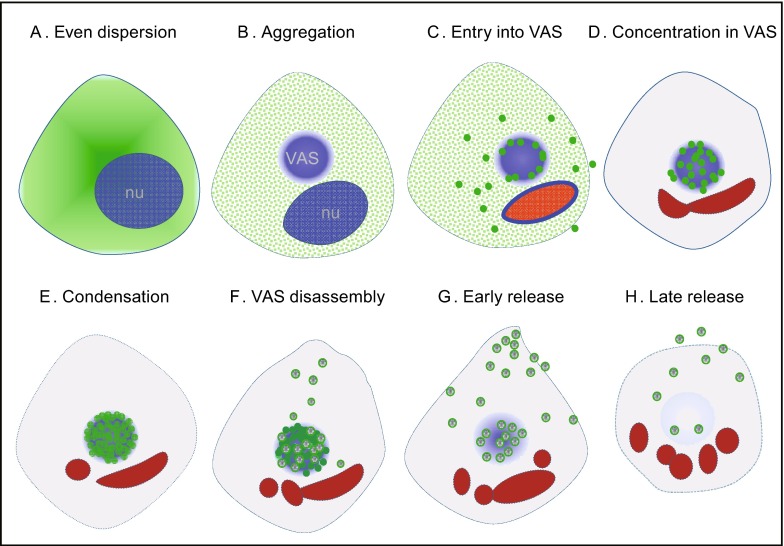
**Representative stages of VAS formation and disassembly**. VP88GFP distributes evenly in the endoplasm. In SGIV-infected cells, VAS forms in a perinuclear area rich in viral DNA, which exhibits strong staining with Hoechst 33342 (blue) as the nucleus (nu). VP88GFP is recruited into VAS and condensed there for assembly and maturation. VAS disassembly occurs when matured virus particles are released out of the host cell. SGIV infection induces host cell death, resulting in nuclear fragmentation and cell membrane leakage permissive to staining by DNA dye PI (red)

We wanted to analyze the subcellular redistribution of VP88GFP in SGIV-infected HX1 cells. To this, VP88GFP-expressing cells were stained with Hoechst 33342 for DNA in the nucleus and VAS, and they were continuously imaged for a period of 6 h starting at 48 hpi with SGIV. This revealed that VP88GFP became fully localized and highly condensed in the VAS within 6 h (Fig. 5). In the same time, the fully developed VAS disappeared as evidenced by a remarkable decrease in its viral DNA content and VP88GFP concentration (Fig. 5H’). The dynamic process of VP88GFP redistribution is more evident in a time-lapse movie (Movie S1A and B). Taken together, VP88GFP shows biphasic redistribution relative to VAS formation and subsequent viral release from VAS, as summarized in schematic diagrams (Fig. 6). Therefore, VP88GFP offers a marker to visualize the redistribution and recruitment of macromolecular components for SGIV assembly and SGIV release.

## DISCUSSION

VAS also known as the “viral factory” is a dynamic cellular structure that forms late during the viral infection cycle and functions in key process of viral replication and/or assembly, and thus represents a target for intervention of viral infection (Novoa et al., 2005; Williams et al., 2005). The VAS of SGIV is a unique subcellular component containing DNA and protein for virus assembly. This DNA gathering point locates at the perinuclear region of the host cell, which can be recognized with DNA staining (Xia et al., 2009; Huang et al., 2013). Understanding of VAS dynamics and underlying mechanisms is pivotal for basic research in host-virus interactions and for the control of viral infection diseases in wild and farmed animals. In this study, we present several independent lines of evidence that the SGIV gene *orf088* offers an excellent molecular marker for visualizing VAS dynamics. First, VP088 expression does not alter the cellular property including an ES cell phenotype, self-renewal, pluripotency gene expression, SGIV susceptibility and host cell response to SGIV infection at molecular and cellular (flow cytometry) levels. Second, VP088 shows subcellular redistribution at various stages of SGIV infection, allowing for real-time visualization of VAS dynamics in a host cell. Finally, real-time imaging reveals that VP088 becomes fully localized to, and condensed in VAS within 6 h, and that fully condensed VP088 disappears together with the viral DNA as a consequence of virion releasing, which establishes, for the first time to our knowledge, a 12-h process for VAS formation and SGIV release in a host cell.

Macromolecular assembly into complexes and cellular structures operates widely in the living system, ranging from viruses to higher eukaryotic organisms including plants and animals. The mechanisms underlying macromolecular assembly in normal and abnormal processes have attracted considerable attention. In this regard, VAS represents an excellent system to elucidate macromolecular assembly, because many, if not all macromolecular components for the VAS formation and ultimate assembly into virions within VAS are of viral origin and thus exogenous to host cells for clear identification. In this study, GFP-tagged VP088 serves an excellent marker for VAS visualization. Prior to SGIV infection, we revealed that GFP-tagged VP088 on itself is a cytoplasmic protein as intracellular expressed VP88GFP distributes evenly in the cytoplasm of a host cell. However, in SGIV-challenged cells, we have demonstrated that VAS formation is initiated before the condensation of VP088. Redistribution of VP88GFP is triggered by viral invasion and exhibits a distinct pattern by condensing in VAS, which is similar to the VAS related distribution of SGIV envelope protein VP19 (Huang et al., 2013), providing direct evidence that this protein is not required for VAS formation. On the contrary, reports indicated the distribution of non-structural proteins encoded by *orf086* or *orf162* has no colocalization with the VAS of SGIV (Xia et al., 2009; Xia et al., 2010). These observations suggest that VP088 is indeed a structural protein of SGIV particles and this notion is also supported by a previous report that the VP088 has three putative transmembrane domains and located as a viral envelope protein (Zhou et al., 2011). A closer inspection leads to a striking finding, which is the redistribution and sequential component recruitment for SGIV assembly in a host cell.

After condensation in the VAS, VP88GFP becomes hardly detectable by fluorescence. This allows for two alternative explanations. One is its degradation after its involvement in maturation. The other is VAS disassembly due to the release of matured virions as its content. We prefer to the second possibility because the disappearance of VP88GFP from VAS accompanies the disappearance of viral DNA and the appearance of the VP88GFP signal in cytoplasmic areas other than VAS. This is also in accordance with VP088 as an envelope protein (Zhou et al., 2011).

Visualization of VP88GFP in this study reveals the dynamic processes of VAS formation and disassembly, which may be described in eight representative stages (Fig. 6). VP88GFP disperses evenly in the cytoplasm, and the SGIV infection procedure does not alter this distribution pattern (Fig. 6A). Upon SGIV infection, VAS formation occurs in the absence of VP88GFP, when VP88GFP undergoes aggregation (Fig. 6B). During subsequent infection, this protein is first seen in the VAS (Fig. 6C), which demarcates the onset of its entry into VAS and suggests sequential recruitment of VP88GFP for SGIV assembly. Meanwhile, the dead cell can be detected by PI staining. When the infection proceeds, VP88GFP becomes concentrated (Fig. 6D) and condensed in the VAS (Fig. 6E). VP88GFP starts to appear outside the VAS (Fig. 6F), suggesting VAS disassembly and virion release into the nearby cytoplasm. Ultimately, VP88GFP-containing SGIV virus particles spread throughout the cytoplasm (Fig. 6G) and finally release out of the cell membrane (Fig. 6H), completing the infectious cycle. The host cells become dead by apoptosis and necrosis as evidenced by PI staining and nuclear fragmentation (Fig. 6D–H).

Viral infection brings about two major events, namely virus propagation and host cell response. SGIV causes host cell death by two pathways: One is non-apoptotic programmed cell death (PCD) as has been reported in its natural host species (Huang et al., 2011), the other is apoptosis as has been reported in non-natural host species and medaka HX1 cells in culture (Huang et al., 2011; Yuan et al., 2013). In this study, we have observed that SGIV induces not only apoptosis as a major death pathway but also necrosis at a detectable level.

More importantly, one interesting observation is that the distribution of ectopic expressed VP88GFP changed after virus infection by aggregation and condensation into the VAS. However, our results here do not reveal the distribution process of viral genome itself encoded VP088 throughout the infection cycle. The gene encoding VP88GFP is inserted into the host genome together with a CMV promoter, but the VP088 is encoded by the genome of the infected virus. The gene copy numbers of them are different, and the expression of each protein is driven by a different promoter. Additionally, the timing of protein expression varied from each other. The VP88GFP is expressed before the virus infection, but the expression of VP088 is activated only after virus infection. Considering the above concerns, generation of a recombinant SGIV containing a GFP-tagged VP088 will resolve this issue in the future. Successful visualization of VAS dynamics with fluorescent protein tagged virus has been reported (Heath et al., 2001). Future work is needed to elucidate the mechanisms underlying programmed aggregation and cell death commencement as well as the mechanism underlying SGIV infection-dependent redistribution of VP088 and the precise role that VP088 plays in SGIV assembly and release. The recently published study has illustrated the assembly and budding of SGIV with electron miscopy (Liu et al., 2016) and the details of how SGIV entry into host cells by labeling the SGIV particles with chemical dye (Wang et al., 2014).

In summary, VP088 is not cytotoxic and does not compromise the ES cell property, viral susceptibility and host-virus interactions. This protein undergoes SGIV-dependent subcellular redistribution and shows sequential recruitment into the VAS for viral assembly. These features make VP88GFP an excellent marker for generating GFP-tagged recombinant SGIV for the experimental analysis and real-time visualization of SGIV infection.

## MATERIALS AND METHODS

### Fish

Work with fish followed the guidelines on the Care and Use of Animals for Scientific Purposes of the National Advisory Committee for Laboratory Animal Research in Singapore and approved by this committee (permit number 27/09). Medaka was maintained under an artificial photoperiod of 14-h/10-h light/darkness at 26°C as described (Li et al., 2009; Hong et al., 2010).

### Plasmids

Plasmid p88GFP that encodes the fusion protein VP88GFP between VP088 and GFP was constructed by three-component ligation. Briefly, the *orf088* coding sequence (CDS) was amplified by using primers orf088Eco (aa*gaattc*accATGGGCGCAGCGC) plus orf088Hind (gc*aagctt*CTTTGCAGCTTC) from SGIV, and the *gfp* CDS was PCR-amplified by using primers GFPHind (gc*aagctt*GTGAGCAAGGGCGAG) plus GFPXho (ga*ctcgag*TCACTTGTACAGCTCG) from pEGFP-N1 (Clontech). The PCR products were digested with *Eco*RI plus *Hin*dIII (*orf088* fragment) or *Hin*dIII plus *Xho*I (*gfp* fragment) and combined with *Eco*RI-*Xho*I double-digested pcDNA3.1 for ligation. Control plasmid pGFP was generated with an insertion gene of *gfp* between restriction sites of *Eco*RI and *Xho*I in pcDAN3.1. Correct constructs were confirmed by sequencing. Plasmid DNA was prepared with a Midiprep kit (Qiagen, Valencia, CA, USA).

### Cell culture and transfection

The medaka haploid ES cell line HX1 was maintained at 28°C in the medium of ESM4 as previously described (Hong and Schartl, 2006; Yi et al., 2010). The grouper spleen cell line GS was maintained at 25°C in L15-medium (Leibovitz) containing 10% fetal bovine serum (Huang et al., 2009). Cell transfection was performed by using DNAfectin reagent (Applied Biological Materials, Richmond, BC, Canada) essentially as described (Hong et al., 2004a). Briefly, 2 µg of plasmid DNA (p88GFP or pGFP) and 8 µL of DNAfectin reagent were mixed in 200 µL of pure DMEM. After incubation at room temperature for 20 min, the transfection mixture was added dropwise to cells in a 6-well plate containing 2 mL of DMEM. After incubation for 6 h at 28°C, the cells were grown in ESM4 for 48 h and subcultured in 10-cm dishes for clonal growth in the presence of 0.5 mg/mL of G418 (Hong et al., 1996). The medium was changed every 5–7 days. Single colonies comprising GFP-positive cells were picked with 200-µL tips into 96-well plates and serially expanded into 88GFP-HX1 cells (p88GFP transfectants) and GFP-HX1 cells (pGFP transfectants) as described (Hong et al., 1996).

### Virus preparation and inoculation

SGIV (strain A3/12/98) originally isolated from the diseased brown-spotted grouper (*E. tauvina*) was propagated in GS cells as described (Qin et al., 2003). Briefly, SGIV was inoculated onto confluent GS cells at a multiplicity of infection (MOI) of ∼0.1. Upon the appearance of apparent cytopathic effect, cells were harvested and centrifuged at 3000 ×*g* for 10 min at 4°C, the cell debris together with partial supernatant were collected and stored at −80°C until use. HX1 cells were infected similarly.

### RT-PCR analysis

RNA isolation from cell culture and RT-PCR analyses were performed as described (Hong et al., 2004b; Yuan et al., 2013). PCR was run in a 20-µL volume containing 10 ng of cDNA reaction for 25 (*β-actin* as a loading control) and 35 cycles (95°C for 30 s, 60°C for 20 s and 72°C for 1 min; other genes). PCR products were separated on 2% agarose gels. Primers used are listed in Table S1.

### Cell growth assay

Cell growth was analyzed as described (Hong et al., 1996; Yi et al., 2009). Briefly, 10^5^ of 88GFP-HX1 and GFP-HX1 cells were seeded into the 6-well plate and counted in triplicates every 24 h until 8 days of culture.

### Cell staining

Growing cells in culture were co-stained with Hoechst 33342 and propidium iodide (PI) before fluorescent microscopic observation. In detail, the culture medium containing Hoechst 33342 (1 μg/mL) plus PI (1 μg/mL) were added carefully into the culture containing virus-infected cells and incubated at 28°C for 10 min. To reduce the fluorescence background, the cells were carefully rinsed in phosphate buffered saline (PBS) and refed with fresh medium. Nuclear staining in living cells (Hoechst 33342) and dying/dead cells (PI) was visualized by fluorescent microscopy.

### Flow cytometric assay

HX1, GFP-HX1 and 88GFP-HX1 cells at 48 hpi with SGIV (MOI of 0.1) were trypsinized into single cell suspension and 10^5^ cells were stained with 5 μL of Annexin V/pacific blue (Invitrogen, USA) in 100 μL of binding buffer for 15 min at room temperature and counterstained with PI at 50 μg/mL. SGIV infected cells and mock control cells were analyzed on the BD LSR Fortessa (Becton Dickinson, San Jose, CA, USA).

### Microscopy

Observation and photography on Zeiss Axiovert invert microscope with a Zeiss AxioCam M5Rc digital camera (Zeiss Corp., Germany) were done as described (Yi et al., 2009; Yuan et al., 2014). Confocal microscopic observation and time-lapse imaging were performed on the UltraView VoX (PerkinElmer, Waltham, MA, USA) using an Olympus water-immersion 40× objective lens (NA = 1.15; Olympus, Tokyo, Japan) by using software Volocity 6.2.1 (PerkinElmer) setting for sequential record modes at 3 channels of laser lines at 405, 488 and 561 nm.

### Statistical analysis

The Dunnett’s test was conducted by using GraphPad Prism v4.0. Data are presented as means ± S.D, and *P* < 0.05 were calculated by using Student’s *t*-test and considered as significant differences as described (Yi et al., 2010).

## Electronic supplementary material

Below is the link to the electronic supplementary material.

Supplementary material 1 (MOV 663 kb)

Supplementary material 2 (JPEG 168 kb)

Supplementary material 3 (JPEG 125 kb)

Supplementary material 4 (PDF 181 kb)

Supplementary material 5 (PDF 113 kb)
