# Supplementary figures and images for: Subcellular redistribution and sequential recruitment of macromolecular components during SGIV assembly
Source: Protein Cell. 2016 Jul 18;7(9):651–61. doi: 10.1007/s13238-016-0292-3 (PMC5003786; doi:10.1007/s13238-016-0292-3)

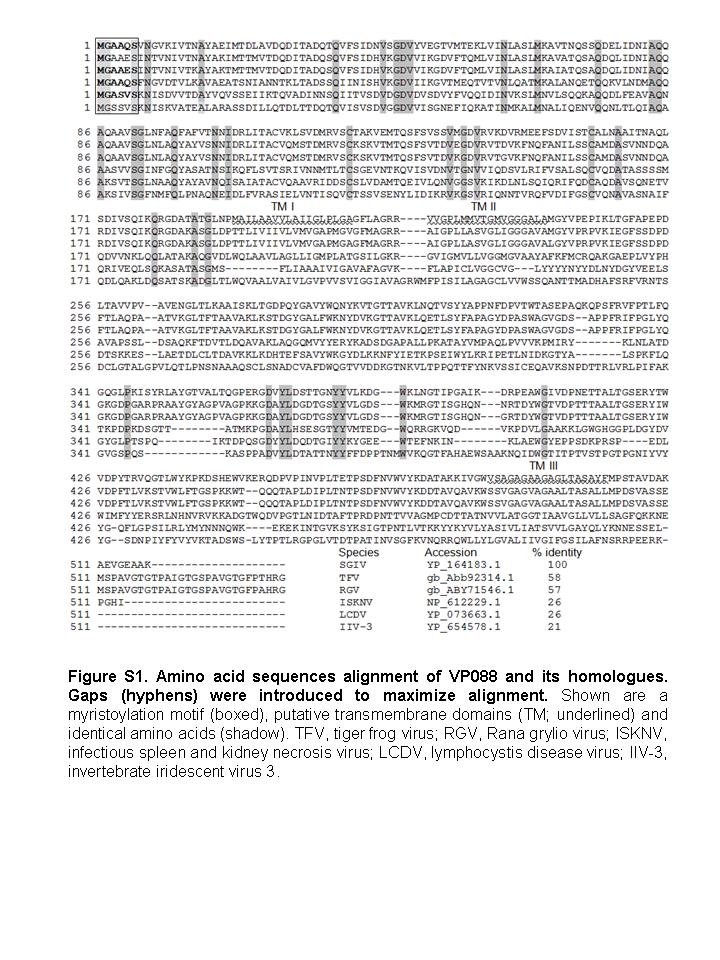

Supplement: Supplementary file 2 — Supplementary material 2 (JPEG 168 kb) [file 13238_2016_292_MOESM2_ESM.jpg]

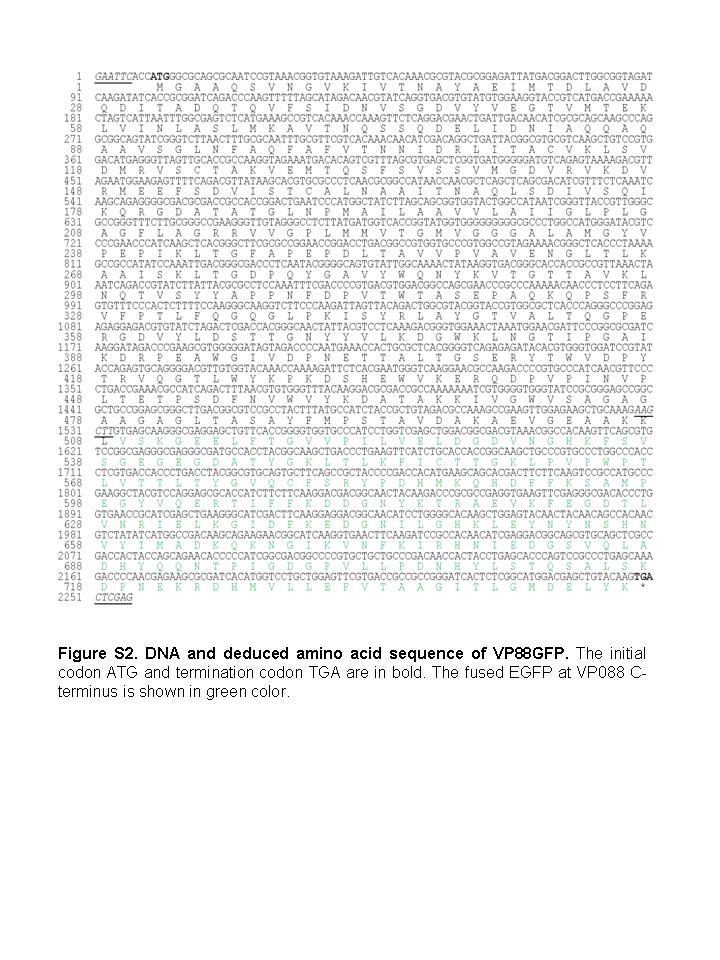

Supplement: Supplementary file 3 — Supplementary material 3 (JPEG 125 kb) [file 13238_2016_292_MOESM3_ESM.jpg]
