# Supplementary material for: Subcellular redistribution and sequential recruitment of macromolecular components during SGIV assembly
Source: Protein Cell. 2016 Jul 18;7(9):651–61. doi: 10.1007/s13238-016-0292-3 (PMC5003786; doi:10.1007/s13238-016-0292-3)
Supplement: Supplementary file 4 — Supplementary material 4 (PDF 181 kb) [file 13238_2016_292_MOESM4_ESM.pdf]

Supplementary materials

Fig. S1. Amino acid sequence alignment of VP088 and its homologs. Gaps (hyphens) were introduced to maximize alignment. Shown are a myristylation motif at the N-terminus (bold), putative transmembrane domains (TM; underlined) and *N*-glycosylation sites (box). Identical positions are highlighted. TFV, tiger frog virus; RGV, Rana grylio virus; ISKNV, infectious spleen and kidney necrosis virus; LCDV, lymphocystis disease virus.

Fig. S2. DNA and deduced amino acid sequence of VP88GFP. The initial codon ATG and stop codon of TGA are in bold. VP088 is in black color and GFP is in green color.

Movie S1. Visualization of VAS dynamics. 88GFP-HX1 cells at 48 hpi with SGIV were stained with Hoechst 33342 for time-lapse observation. Behaviors of nuclei plus viral DNA (blue) and VP88GFP (green) are shown during VAS formation and disappearance in the split channel.

Table S1. Genes and primers used for RT-PCR analyses.
