# Supplementary material for: Subcellular redistribution and sequential recruitment of macromolecular components during SGIV assembly
Source: Protein Cell. 2016 Jul 18;7(9):651–61. doi: 10.1007/s13238-016-0292-3 (PMC5003786; doi:10.1007/s13238-016-0292-3)
Supplement: Supplementary file 5 — Supplementary material 5 (PDF 113 kb) [file 13238_2016_292_MOESM5_ESM.pdf]

Table S1. Genes and primers used for RT-PCR analyses

Gene	Accession number	Primer	Sequences (5' to 3')	Size (bp)
orf088	AAS18103.1	ORF088F	ATGGGCGCAGCGCAATC	520
		ORF088R	CGATGTCGCTGAGCTG	
oct4	AY639946	oct4F	GTAGGTCACCTGACAGGATG	660
		oct4R	CTGATTGCACTCTGACAGC	
nanog	FJ436046	nanogF	ATGGTTGAGTCCCAATC	321
		nanogR	ATATCGCTCTGAAACCCAG	
brachyury (ntl, no tail)	ENSORLG00000011262	ntlF	CTGCCTACCAGAACGAAGAGA	985
		ntlR	TTCGATCAGTAGAAGGCACGT	
β-actin	S74868	ActinF	TTCAACAGCCCTGCCATGTA	650
		ActinR	CCTCCAATCCAGACAGTAT	
